# Mycotoxin Contamination in Smallholder Maize Production: Farmers’ Perceptions, Control Practices, and Influencing Factors in South Africa

**DOI:** 10.3390/toxins18070289

**Published:** 2026-06-30

**Authors:** Steven Sifiso Shange, Temitope Oluwaseun Olorunfemi, Oluwasogo David Olorunfemi

**Affiliations:** 1 Faculty of Agriculture and Natural Sciences, School of Agricultural Sciences, University of Mpumalanga, Private Bag X11283, Mbombela 1200, South Africa; dtanalyticsconsults@gmail.com; 2 Eduvos, Mbombela Campus, Mbombela 1200, South Africa; temitope.olorunfemi@eduvos.com

**Keywords:** mycotoxin contamination, smallholder maize farmers, food safety, agricultural extension, prevention and control practices, South Africa

## Abstract

Globally, mycotoxin contamination of maize is a fundamental concern due to significant economic losses and toxic health effects on humans and animals. This study analyses the perceived effects of mycotoxin contamination and the use of control measures among smallholder maize farmers in South Africa using Mbombela as a case study. A two-stage sampling procedure was used to select 152 registered smallholder maize farmers in Mbombela, South Africa. Data was collected with a structured questionnaire administered by trained enumerators. Descriptive and multiple linear regression analyses were carried out using SPSS (Version 28). The findings revealed that many farmers had a high perception of the effects of mycotoxin contamination, and the most prominent prevention and control practices were good field management, storage of maize in clean, well-ventilated stores, and proper sorting of harvested grains. Multiple linear regression results revealed that farming experience, media exposure, extension visit, mycotoxin-related training, mycotoxin awareness, and perception index significantly influenced farmers’ utilization of mycotoxin prevention and control practices. The study recommended that agricultural professionals develop robust mycotoxin-related training and advisory services to enhance and strengthen farmers’ awareness and perceptions, and to promote the sustained use of effective agricultural practices to combat mycotoxin contamination.

## 1. Introduction

Globally, the contamination of food and feed crops by mycotoxins is a significant concern [1] and has led to reduced maize production in several countries, including South Africa [2]. Smallholder maize farmers in Africa face considerable challenges with mycotoxin contamination, particularly aflatoxins [3]. These mycotoxins are small, stable natural compounds that accumulate in maize crops following fungal infections during crop development, harvesting, and post-harvest storage [4]. Factors such as insect and pest damage, plant vulnerability, and ecological conditions contribute to the fungal infections that produce mycotoxins [2]. Additionally, weather variability, inadequate storage infrastructure, poor agricultural practices, and limited knowledge about mycotoxins place many farming communities at high risk of exposure [5]. Contaminated maize poses serious risks, including harmful effects on humans and animals, leading to health issues and financial losses [2]. Consequently, mycotoxin contamination of maize is a pressing issue that demands attention due to its substantial economic impacts and toxic effects on both humans and animals. Chukwudi et al. [6] emphasized that maize cultivation in South Africa faces various abiotic and biotic challenges, including nutrient shortages, drought, pests, and diseases, all of which lower yields and crop quality. However, mycotoxins are among the most significant biotic stressors affecting the yields of crops such as maize, sorghum, millet, and wheat in South Africa [7].

Maize is the staple crop in South Africa, with about 10 million tons produced annually by both commercial and smallholder farmers, who cultivate about 250,000 hectares [8]. It is the most important crop and primary food source for many rural households in South Africa, contributing to poverty reduction, food security, income, and community livelihoods [9]. Its consumption is expected to increase by 2027 due to the growing human and animal populations [6]. Nonetheless, it remains one of the most susceptible cereal crops to mycotoxin contamination [10]. According to Ayelign and De Saeger [11], maize crops are exposed to fungal secondary metabolites, known as mycotoxins, which pose serious health risks to humans, because they can persist in food products and enter the food chain, causing acute and chronic disorders such as liver and kidney damage, immune suppression, stunted growth in children, and an increased risk of cancer and contributing to economic decline, hunger, and malnutrition. Also, in animal nutrition, the consumption of mycotoxin-contaminated maize can lead to growth impairment, kidney enlargement, hepatocellular hyperplasia, cirrhosis, and acute liver damage in chickens, pigs, and dairy cattle [12], ultimately resulting in significant economic losses in livestock production. Consequently, these maize contaminants exacerbate global concerns, particularly in smallholder farming in developing countries, about the impact of mycotoxins on agricultural markets, adversely affecting crop yields, livestock production, farmers’ livelihoods, health, and food safety, as well as the costs associated with mycotoxin regulatory programs and technologies [13]. Furthermore, the public health risks posed by mycotoxin contamination, arising from a lack of understanding in various regions of Africa, including South Africa, represent a knowledge gap that requires further attention for effective management measures [14]. Misihairabgwi et al. [15] reported that smallholder farmers in South Africa have limited knowledge of mycotoxins, thereby increasing their vulnerability and risk. This underscores the need for greater focus on mycotoxin research, particularly among smallholder maize farmers.

High levels of mycotoxin contamination have been identified in the provinces of KwaZulu-Natal, Limpopo, Eastern Cape, and Mpumalanga [16], which have both direct and indirect impacts on smallholder farmers’ livelihoods, food security, and safety in these areas [17]. Moreover, recent studies have identified Mpumalanga Province as having a high prevalence of maize mycotoxin contamination. For instance, a study conducted by Chukwudi et al. [6] revealed that Mpumalanga Province has the highest concentrations of deoxynivalenol (DON) and zearalenone (ZEA) in maize samples. A critical review of the literature observed a dearth of research on smallholder farmers’ perceptions regarding mycotoxin contamination and their use of control measures in the province. According to Phokane et al. [18], agricultural extension services are well-positioned to play a pivotal role in assisting smallholder farmers by providing information, training, and advisory support to implement effective control measures to mitigate the effects of mycotoxin contamination. Thus, this empirical study is pertinent for providing them with adequate insights and information on effective strategies to ensure that smallholder farmers are well-informed about the consequences of mycotoxins and can implement improved farming practices and safety measures in their maize production, thereby reducing economic and health risks.

This study investigated the perceived effects of mycotoxin contamination among smallholder maize farmers and their use of control measures, focusing on Mbombela in Mpumalanga Province, one of South Africa’s provinces with a high prevalence of maize mycotoxin contamination. The study examined smallholder maize farmers’ perceptions of how mycotoxin contamination affects their income, health, and livelihoods. It also explored the agricultural practices these farmers currently employ to prevent and control mycotoxin contamination in maize production, identified the challenges they face in these efforts, and analyzed the socio-economic factors influencing their use of mycotoxin prevention and control methods.

## 2. Literature Review and Conceptual Framework

In many developing countries, including rural South Africa, concerns about household food security often outweigh concerns about food safety, leaving several rural households vulnerable to food contamination [19]. Maize is a major crop and staple food in South Africa, helping to alleviate poverty and ensure food security, and it is primarily grown by smallholder farmers in rural areas [20]. Unfortunately, maize is prone to mycotoxin contamination, compromising its quality and negatively affecting farmers’ health, income, and livelihoods. According to Gnonlonfin et al. [21], mycotoxin contamination significantly impacts health and crop value in Africa, and high levels in maize consumed by smallholder farmers in South Africa pose a significant health risk [9]. Mycotoxins, which are harmful chemical substances produced by moulds and are invisible, odourless, and tasteless, can contaminate crops in the field or during storage [22]. Mycotoxin varieties include fumonisins, zearalenone, trichothecenes, patulin, aflatoxins, and ochratoxins, and the World Health Organization (WHO) has listed some of these as human carcinogens [23]. Nonetheless, practically all mycotoxins have the potential to cause one or many health issues. Therefore, the safety of maize food and feed components is a global challenge [24], as the occurrence of mycotoxins is a worldwide concern [25].

Given the known socio-economic and health impacts of mycotoxins on African livelihoods, effective and sustainable mycotoxin prevention and mitigation strategies should be established. In South African rural areas where technologies are limited and complex, simple, cultural, acceptable, practical, economically viable, and sustainable measures of mycotoxin control are important in smallholder farming [9]. Apparently, following good agricultural practices, such as sorting grains before storage and crop rotation, can minimize the risk of mycotoxin contamination of maize in smallholder farming systems [17]. Other important practices, including proper pre- and post-harvest handling, are recommended to reduce mycotoxin contamination. Furthermore, Misihairabgwi et al. [26] suggest using technology and community-based methods, improved maize varieties, dietary diversity, and mycotoxin legislation and regulations in Southern Africa to mitigate mycotoxin contamination and its impacts. Thus, awareness and knowledge of mycotoxin contamination, its risks, and its control from production to consumption are vital to effectively manage mycotoxin occurrence and effects.

Phokane et al. [17] identified a lack of knowledge among smallholder farmers about crop storage and the health risks associated with mycotoxin contamination as a major concern. They indicated that farmers store poorly drained crops at high temperatures, which promotes the growth of mycotoxigenic fungi, reducing crop quality and leading to crop losses. This indicates that a lack of awareness and knowledge puts farmers and communities at high risk of consuming mycotoxin-contaminated maize and maize products, which may pose health risks [15]. Also, farmers without knowledge of mycotoxins are unwilling to invest in control measures against mycotoxin contamination, especially smallholder farmers who depend on informal markets with weak regulations [26]. Furthermore, South African smallholder maize farmers face structural constraints, including poor packaging materials and storage facilities, as well as a lack of markets and market access for their produce [27]. This also significantly influences the occurrence of mycotoxins in maize crops among smallholder farmers, raising food safety concerns. Also, according to Chingala et al. [28], farmers’ socio-economic factors influence their awareness and are important in determining their coping strategies. Socio-economic factors that may negatively influence smallholder perceptions include farmers’ access to information, education, income, age, and gender [28].

**Figure 1:**
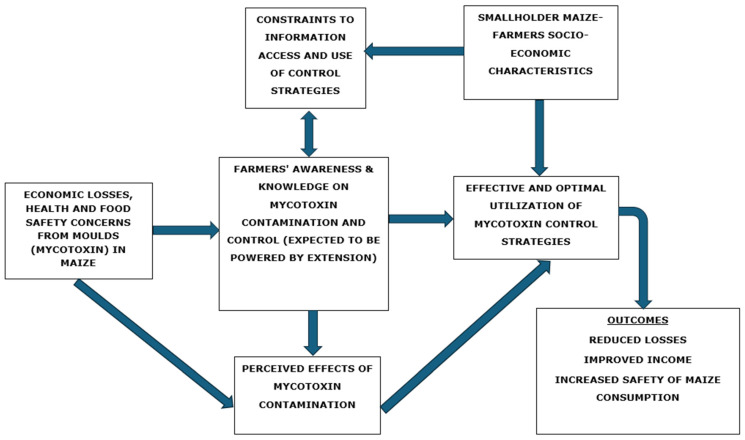
Conceptual framework on farmers’ perceived effects of mycotoxin contamination and utilization of control practices among maize farmers.

Based on a synthesis of the reviewed literature, a framework (Figure 1) was developed to illustrate the interactions among the variables measured in the study and how these interrelationships influence the use of mycotoxin prevention and control practices in maize farming. This framework addresses the economic losses and safety concerns resulting from mycotoxin contamination in maize. It demonstrates that the socioeconomic profile of smallholder maize farmers, including age, educational level, farming experience, farm size, and exposure to training, can directly affect their utilization of mycotoxin prevention and control practices. Additionally, this socioeconomic profile can influence the constraints farmers face in accessing information and coping with mycotoxin contamination and control. Moreover, farmers’ awareness and knowledge of mycotoxin contamination shape their perceptions of its effects on their production and livelihoods. A farmer’s perception of mycotoxins also affects the adoption and use of control strategies to mitigate the health, income, and livelihood impacts of contamination. When farmers are aware of mycotoxin contamination and have a positive perception of its impacts, they are more likely to effectively adopt and utilize recommended control strategies from extension officials and other agricultural stakeholders. Ultimately, increased utilization of mycotoxin prevention and control strategies by smallholder maize farmers is expected to lead to improved income, reduced losses, and enhanced safety in maize consumption.

## 3. Results and Discussion

### 3.1. Socio-Economic Characteristics of the Maize Farmers

The findings in Table 1 reveal that the mean age of the maize farmers was 45.61 years. This implies that most of the smallholder maize farmers in Mbombela were middle-aged and are potentially expected to be productive, innovative, with a high level of information-seeking behaviour, and have a high degree of exposure as it relates to mycotoxin contamination and control in maize production. Also, more than half of the maize smallholder farmers were females (56.6%). Generally, farming is considered a male-dominated occupation because it often requires manpower; males are naturally stronger than females and culturally have greater access to land [29]. Nevertheless, the dominance of female farmers in the study area might be a result of the observed fact that several households in the area are female-headed, coupled with the fact that a lot of men in the area are now often going for paid jobs as opposed to the women who spend most of the time at home in the community doing household chores. As a result, women tend to practice subsistence farming for consumption close to their households. Mostly, they have backyard gardens, and some have plots in the communal farmlands where they plant maize. Furthermore, more than half (52.6%) of the respondents were not married. These included the widowed, divorced, and never married individuals. This finding is contrary to Agbugba et al. [27], who reported that in the Eastern Cape province of South Africa, most maize farmers were married. The unmarried respondents in the study area are expected to be free of some marital-related responsibilities, which might allow them more time to socialize and seek up-to-date information on their maize-based enterprise, such as mycotoxin contamination and control from other farmers and groups.

Moreover, Table 1 shows that the mean household size among the farmers was 6 people. This implies that, on average, most farmers in the area have the potential to involve household members in their maize farm activities to reduce external labor costs. Furthermore, large households tend to be exposed to a wider range of information sources and have greater potential for information sharing among members. This could likely affect farmers’ awareness in such households of burning issues such as mycotoxin contamination and its control in their maize enterprise. The results in Table 1 show that the majority (97.4%) of the maize farmers in the study area had some years of formal education, with the mean years of formal education in the study area being 10.25 years. As such, these farmers are more likely to be aware of mycotoxin contamination, including the risks associated with contamination and the control measures. This finding differs from Myeni et al. [30], who reported that the majority of smallholder farmers in South Africa have limited education, but this is not the case among Mbombela maize farmers. Table 1 further reveals that about one-third (31.6%) of the farmers had between 1 and 5 years of maize farming experience, 29.6% had between 6 and 10 years, and 38.8% had more than 11 years of maize farming experience. The mean years of maize farming experience among the smallholder farmers is 11.41 years. This indicates that the maize farmers have some level of experience spanning over a decade in maize cultivation. This is similar to the findings of Olorunfemi et al. [31] and Otekhile and Verter [32], who reported in their respective study that maize farmers in Nigeria had an average age of between 11 and 13 years. Generally, experience generates knowledge and awareness; it is expected that, on average, maize farmers in the area are aware of mycotoxin contamination, as some would have experienced its effects on their maize production over the years.

Also, Table 1 shows that the majority (77.6%) of the maize farmers indicated that they do receive visits from extension agents. This implies that many respondents are expected to have access to professional agricultural information, training, and advice on agricultural practices, such as mycotoxin contamination and related topics, that could improve their income and enhance their livelihoods. According to Oduniyi et al. [33], access to extension services influences awareness and, consequently, the adoption of sustainable agricultural practices. Furthermore, the study findings show that majority (79.6%) of the maize farmers indicated that they have not been exposed to any mycotoxin-related training. This implies that maize farmers in the study area are likely to have minimal knowledge of mycotoxin contamination and control, despite the high rate of extension visits. This indicates that extension programmes offered to the farmers in the area are still lacking in information relating to mycotoxin contamination awareness and control, and this needs to be improved upon by extension stakeholders in the area. According to Table 1, the mean farm size allocated to maize farming in the area is 1.31 ha. This implies that the majority of smallholder farmers are smallholders who mainly plant for household consumption and possibly sell excess in informal markets.

Table 1 shows that although some farmers (46.7%) in Mbombela District were members of a farmer group, more than half (53.3%) of the maize farmers did not belong to any farmer group. This implies that these categories of farmers will not be able to leverage the advantages of information sharing and social networking that membership in a group can offer. The study’s findings contrast with those of Oduniyi et al. [33], who reported that the majority of maize farmers in Gert Sibande District, Mpumalanga Province, are members of farmer groups/agricultural cooperatives. Generally, farmers in the farming group have the opportunity to obtain more information on mycotoxins and to share experiences with other maize farmers in the group. Also, farmers who belong to the farm association are more likely to receive extension services, training, and programmes offered by extension organizations to improve their capacity and are thus more easily facilitated in group forums than directly to individual farmers [34]. Such programmes may be related to mycotoxin contamination and management in maize production. Thus, there is a need to sensitize more farmers in the area to the benefits of social networking, collaborative effort, and action. Moreover, the majority (79.6%) of maize farmers are exposed to 3 or more media outlets through which they can access information. This implies that despite the lack of specific training on mycotoxin as indicated in earlier findings, the maize farmers’ exposure to some media outlets, such as television, radio, internet, social media, and published information, has the potential of exposing them to some vital information on improved farm practices, which may include mycotoxin-related information. The high literacy level among farmers could also enhance their information-seeking behavior through these outlets. Ekwomadu et al. [35] recommended that the proper use of media outlets by extension and rural advisory stakeholders could lead to effective education for farmers, traders, and consumers on mycotoxins, their effects, and the various strategies to control their occurrence.

### 3.2. Perceived Effects of Mycotoxin Contamination by Smallholder Maize Farmers 

Using the mean score to rank the perceived effects statements relating to maize mycotoxin contamination, the results in Table 2 reveal that smallholder farmers had a positive perception of the inimical effects of maize mycotoxin contamination, as the mean score for all statements was above the benchmark of 3.0. The most prominent statements on mycotoxin contamination effects which were positively perceived by farmers in the area were ‘Mycotoxin contamination will lead to the reduced market price of maize’ (MS = 4.43), ‘Mycotoxin contaminated maize will be difficult to sell’ (MS = 4.41), ‘Spoilt maize should not be sold or sent to the markets’ (MS = 4.36), ‘Mycotoxin contamination often affect the change in taste, smell and colour of maize grains’ (MS = 4.28), ‘Mycotoxin maize contamination may lead to household income loss’ (4.25), and ‘Mycotoxin contamination in maize is an important health hazard’ (MS = 4.25). The results reveal that most farmers are aware of and agree that mycotoxin contamination in maize may reduce maize market prices, as this statement ranked first. This finding is consistent with Gichohi-Wainaina et al. [36], who reported that smallholder farmers in Malawi demonstrated high knowledge and perceptions of the effects of mycotoxin contamination on income loss. Further engagements with the farmers during data collection reveal that when their maize products are contaminated, they are difficult to sell, and farmers decide not to sell or send contaminated maize to the market at all. This causes farmers to experience household income losses due to maize contamination. This is because farmers understand mycotoxin contamination often affects the change in taste, smell, and colour of maize grains. As a result, farmers receive low returns on their maize investments, which contribute to high poverty levels, food insecurity, and hunger. In a bid to survive, farmers sometimes consume mycotoxin-contaminated foods [37]. In addition, the farmers exhibited a high perception that mycotoxin-contaminated maize poses health hazards. Similarly, Mboya and Kolanisi [38] conducted a study to explore smallholder farm households’ awareness of the potential effects of consuming fungal-infected maize in Rungwe district and concluded that farm households perceived potential human health risks associated with consuming fungal-infected crops. According to Shaphard [39], mycotoxin contamination of food has been linked with the development of a wide range of chronic diseases in humans and animals.

Other less prominently perceived statements on mycotoxin contamination effects among the smallholder maize farmers were ‘consumption of mycotoxin contaminated maize can result in a delay in child growth’; ‘resistance of animals to diseases can be reduced by the consumption of mycotoxin contaminated maize’; ‘the use of spoilt maize in making traditional beer should be avoided’; ‘contaminated maize should not be used as animal feed’; ‘the use of mycotoxin contaminated maize by humans can result in mild to severe disease complication such as cancer’; and ‘the adverse health effects of mycotoxins range from acute poisoning to long-term effects such as immune deficiency and cancer’. As reported by Gichohi-Wainaina et al. [36], smallholder farmers in Malawi still exhibited relatively high knowledge of the effects of mycotoxins on children’s growth and development and immunity. However, farmers in the study area still seem to have a lower perception of these issues than of other perceived effects of mycotoxins. Thus, although overall, a high perception level of the effects of maize mycotoxin contamination was observed among the maize farmers in the study area, more sensitization and education campaigns will still be required by extension organizations in the area to increase their knowledge level on some of the less prominently perceived statements on the effects of mycotoxin contamination.

### 3.3. Smallholder Maize Farmers’ Utilization of Mycotoxin Preventive and Control Practices

**Figure 2:**
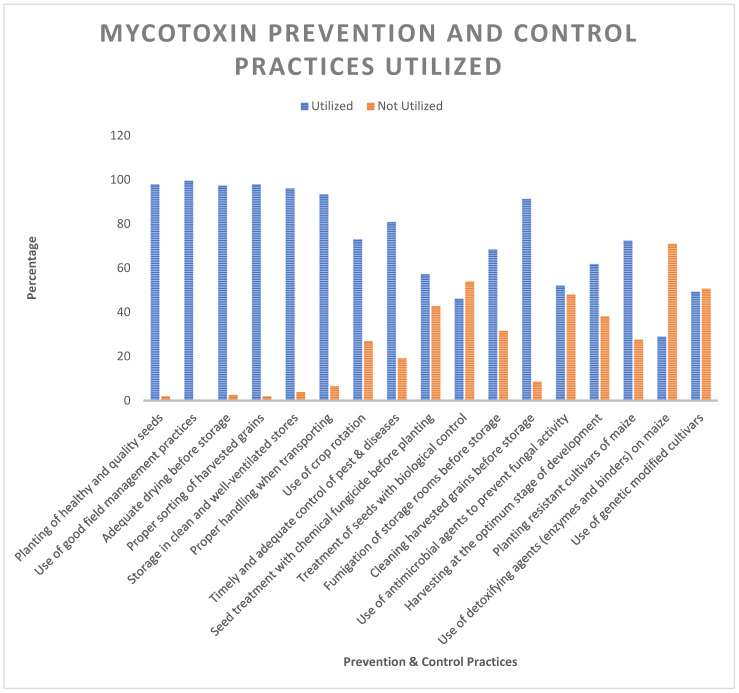
Utilization of mycotoxin preventive and control practices.

Figure 2 reveals that the prominent prevention and control practices utilized were the use of good field management practices (99.7%), storage of maize in clean and well-ventilated stores (96.1%), planting of healthy and quality seeds (98.0%), adequate drying before storage (97.4%), and proper sorting of harvested grains (98.0%). These results agree with Xu et al. [40], who recommended that practical good agricultural practices for smallholder farmers include using drought-tolerant varieties, timely harvesting, proper drying, and suitable storage conditions. On the other hand, the smallholder maize farmers exhibited a low use of the following mycotoxin preventive and control practices, such as ‘use of antimicrobial agents to prevent fungal activity on maize’, ‘removal of stubble from previous crops’, ‘use of genetic modification cultivars’, ‘seed treatment with chemical fungicide before planting’, ‘harvesting maize at the optimum stage of development’, and the ‘use of detoxifying agents (enzymes and binders) on maize’. The low use of these preventive and control strategies by maize farmers may be due to their limited knowledge of these technologies or their inability to access the inputs required to apply them effectively. However, not all potent farm technologies are available to or accessible for resource farmers in developing countries. According to Hell and Mutegi [41], some farmers may not utilize certain strategies not because they are unaware of mycotoxins and/or their effects, but because they do not know the available technology, or because the technology is labor-intensive or costly. These findings suggest that while smallholder maize farmers widely utilize basic and affordable mycotoxin prevention and control measures, there remains limited adoption of more advanced preventive technologies that could further reduce contamination risks. In the context of resource-constrained smallholder farming systems, priority should be given to promoting integrated mycotoxin management approaches that are practical, affordable, and locally adaptable. These include the use of certified disease-resistant and drought-tolerant maize varieties, timely harvesting at physiological maturity, crop rotation, removal of infected crop residues, proper seed treatment, and improved post-harvest handling and storage technologies such as hermetic storage bags and moisture monitoring systems. To improve the current situation, agricultural extension services, research institutions, and government stakeholders should intensify farmer training and awareness programmes on mycotoxin risks and available control technologies, while facilitating access to affordable quality inputs through subsidies, farmer cooperatives, and credit support schemes.

### 3.4. Challenges Faced by Smallholder Maize Farmers in Prevention and Control of Mycotoxin Contamination

Table 3 presents findings on the constraints maize farmers face in controlling mycotoxin contamination on their farms. From the mean rankings, inadequate awareness and training on mycotoxin contamination and control, lack of access to adequate information on prevention and control, and the high cost of improved seeds and mycotoxin-resistant maize cultivars ranked first, each with a mean score of 2.50. This implies that these three were the topmost severe constraints affecting smallholder maize farmers’ ability to adequately and effectively prevent and control mycotoxin contamination in the area. Imade et al. [42] also observed similar findings in Nigeria and concluded that, in low- and middle-income countries, information and awareness about mycotoxins are inadequate. This implies that smallholder maize farmers and consumers in the study area will not be able to implement well-informed strategies to prevent and control mycotoxin contamination until they are well educated about mycotoxin effects and the full range of management strategies.

Furthermore, the smallholder maize farmers also indicated inadequate information and training on pest and disease management, credit, access to chemical seed treatment, irrigation technology, soil management, good agricultural practices, and market access as constraints affecting their ability to adequately and effectively prevent and control mycotoxin contamination in the area. This may indicate the need to scale up agricultural extension support and services in the study area. As opined by Mukanga et al. [43], the lack of adequate extension support not only affects the implementation of measures to control mycotoxins but also evidences an information gap in the agricultural extension delivery system.

However, the lack of weed management in the fields (MS = 1.84) ranked 14th and was below the benchmark of 2.0, indicating that this is not a severe challenge for smallholder farmers in preventing and controlling mycotoxin contamination in the area. Smallholder farmers usually use available family labour to control weeds on their farms. Most of the farmers in the study area were aware of the importance of controlling weeds in their maize plots. Similarly, Laizer et al. [44] noted a similar trend among farmers in northern Tanzania, where most were aware of the importance of managing weeds on their farms.

### 3.5. Socio-Economic Determinants of Farmers’ Utilization of Mycotoxin Prevention and Control Methods

The results of the multiple linear regression analysis in Table 4 indicate that the F-test statistic was 4.29 and the *p*-value was <0.01. In addition, the test for multicollinearity among the variables was conducted using the variance inflation factor (VIF); the mean VIF was 1.22, with high tolerance values across the variables, indicating that multicollinearity was not a problem in the model. Furthermore, 6 of the 13 variables included in the model were found to be statistically significant factors influencing the utilization of mycotoxin prevention and control practices among maize farmers in the study area. The results of the multiple linear regression indicated that the coefficients for maize farming experience and extension visits are significant at 10%, while the mycotoxin-related training and awareness score is significant at 5%, and the media exposure and perception score is significant at 1%. This means that these contextual socio-economic factors significantly influenced farmers’ levels of utilization of mycotoxin-contamination prevention and control practices in the study area.

The coefficient for respondents’ maize farming experience was negative (−0.0919496) and significant (*p* < 0.10), indicating a negative influence on farmers’ utilization of mycotoxin prevention and control practices. This implies that the farmer’s experience is likely to affect the farmer’s ability to adopt and implement available mycotoxin prevention and control measures. These results indicate that greater years of maize farming experience among respondents are associated with lower use of mycotoxin prevention and control practices. This might be because greater years of farming experience are often associated with increased age, and older farmers tend to be less innovative than younger ones, which sometimes limits the adoption and use of innovative techniques. In contrast, Akter et al. [45] argue that farming experience has a positive influence, finding that the probability of adopting improved maize seed and pest control strategies in Nigeria increases with farmers’ experience.

Also, the coefficient for media exposure was negative (−0.9578758) and significant (*p* < 0.01), indicating a negative influence on smallholder maize farmers’ utilization of mycotoxin prevention and control strategies. This indicates that, in the study area, increased media exposure has not translated into increased use of mycotoxin control practices. This does not conform to prior expectations, as ideally, increased media exposure would allow farmers to access more vital information on improved farm practices. According to Bhabhor et al. [46], increased media exposure among Tribal Rabi maize growers in India was positively and significantly correlated with higher levels of technology adoption. However, this does not seem to be the case in the study area, as the majority of the media outlets to which farmers are exposed do not provide information on mycotoxin-related issues or control practices. This is evident in the farmers’ pointing out inadequate access to information on the prevention and control of mycotoxins as a severe constraint in the area. Ekwomadu et al. [35] opined that the proper use of media outlets by extension and rural advisory stakeholders could lead to effective education for farmers, traders, and consumers on mycotoxins, their effects, and the various strategies to control their occurrence.

Moreover, the parameter for extension visits had a positive (1.970348) and significant (*p* < 0.10) effect on maize farmers’ utilization of mycotoxin prevention and control methods. This implies that farmers who have more frequent extension visits have a higher potential of effectively utilizing mycotoxin prevention and control practices. This indicates the positive role extension professionals play in providing information that influences the transfer of agricultural innovations and practices to farmers. Extensionists play a significant role in providing specialized agricultural advisory services, which may promote the adoption and implementation of mycotoxin management strategies in maize fields. Adeagbo et al. [47] stated that extension visits positively and significantly influence maize farmers’ adoption of farm adaptation strategies.

Furthermore, the coefficient for mycotoxin-related training was positive (2.39684) and significant (*p* < 0.05) in influencing the utilization of maize farmers’ mycotoxin prevention and control strategies. This indicates that the adoption of mycotoxin prevention and control methods increases as more mycotoxin-related training is provided to farmers. Providing mycotoxin-related training equips farmers with the knowledge to understand mycotoxins in maize, their effects, and possible management strategies. The study’s findings align with those of Sitoe et al. [3], who found that increased farmers’ training positively influences farmers’ innovativeness, efficiency, and use of mycotoxin prevention and control technologies.

Also, the maize farmers’ mycotoxin awareness index had a positive (0.3399412) and statistically significant (*p* < 0.05) influence on the farmers’ utilization of mycotoxin prevention and control methods. This implies that when farmers are more aware of mycotoxin contamination and its effects, their adoption and continued use of mycotoxin prevention and control practices increase. This agrees with Udomkun et al. [37], who reported that farmers’ awareness level significantly influenced their adoption of mycotoxin (aflatoxin) prevention and control practices. Arguably, farmers in regions with historical mycotoxin outbreaks had better maize-handling practices due to the higher level of awareness they had developed from past contamination experiences. Furthermore, data from interviews and surveys in South Africa revealed that people consumed mycotoxin-contaminated staples, suggesting they are not fully aware of the health risks associated with mycotoxin ingestion [38]. Any reduction strategy is hampered by a lack of effective and sustained awareness and education about the threat of mycotoxins to human health [9].

Lastly, the coefficient for the farmers’ perceived effect index was positive (0.1590107) and significant (*p* < 0.01) in its influence on farmers’ utilization of mycotoxin prevention and control practices. A high level of farmers’ perception of the effects of mycotoxin contamination will lead to increased adoption and use of mycotoxin prevention and control practices. As farmers perceive health risks from consuming contaminated crops, they may consider preventive measures in their production systems [37]. Similarly, a study conducted by Elemasho et al. [48] in Nigeria reported a significant relationship between farmers’ perception and their adoption of pre- and post-harvest strategies.

## 4. Conclusions and Recommendations

Global mycotoxin contamination of maize is a fundamental concern that requires continuous attention due to its important commercial losses and toxic health effects on humans and animals. The study concluded that smallholder maize farmers had a high perception of the effects of mycotoxin contamination on human and animal health, as well as on their livelihoods. They employed basic and affordable crop management practices such as proper drying, grain sorting, quality seed selection, and improved storage practices to prevent and control contamination, thereby avoiding income losses and health risks associated with maize contamination. But adoption of more advanced preventive technologies remains limited, which could further reduce contamination risks. Significant barriers that farmers face in effectively preventing and controlling maize contamination on their farms include inadequate mycotoxin-related training, the high cost of improved seeds, and limited agricultural extension support services. Also, farming experience, extension visits, mycotoxin-related training, media exposure, farmers’ awareness index, and perception index were significant factors influencing the farmers’ utilization of mycotoxin prevention and control practices in the study area. These findings and conclusions have implications for upscaling mycotoxin awareness campaigns and capacity-building initiatives for smallholder farmers in the area. In resource-constrained smallholder farming systems, the priority should be to promote integrated mycotoxin management approaches that are practical, affordable, and locally adaptable. These include using certified disease-resistant and drought-tolerant maize varieties, timely harvesting at physiological maturity, crop rotation, removal of infected crop residues, proper seed treatment, and improved post-harvest handling and storage technologies, such as hermetic storage bags and moisture-monitoring systems.

Therefore, it is recommended that agricultural extension professionals and other relevant stakeholders develop a robust mycotoxin-related training and advisory service to enhance farmers’ perceptions and upscale the use of effective agricultural products and practices to combat mycotoxin contamination. Furthermore, strengthening public–private partnerships to improve the availability of resistant cultivars, biological control products, and climate-smart storage technologies will enhance farmers’ capacity to prevent mycotoxin contamination. This will ensure the safety of maize consumption and reduce economic losses among maize farmers. Also, the government should facilitate subsidy programs to help reduce the cost of mycotoxin contamination prevention and control practices, thereby improving accessibility and utilization by farmers. Furthermore, credit facilities and specially packaged soft loans for smallholder farmers should be made available to enhance their financial capacity to increase farm production and to utilize innovation to prevent and control mycotoxin contamination. Extension and rural advisory stakeholders should ensure that their education and farmer capacity-building campaigns include food safety and health issues, such as mycotoxin contamination. Moreover, they should make use of various possible channels and media outlets to make sure such information gets through to the farmers.

## 5. Study Limitations and Future Research Directions

While this study produces promising results, it is important to acknowledge its limitations, as is common in research. Due to time and logistical constraints, the focus was exclusively on maize, the primary staple crop grown by smallholder farmers in the study area, which is particularly vulnerable to mycotoxin contamination. Furthermore, the research was limited to smallholder maize farmers in Mbombela Municipality, and only registered farmers were selected at the time of the study. The study used a survey questionnaire, which assumes respondents provide accurate information. To enhance response accuracy, techniques such as rephrasing questions to capture essential information and reduce response errors were employed. Additionally, the researcher drew on prior knowledge that each type of mould present in maize differs slightly in colour, aiding in the identification of prevalent fungal infections. During the research and data collection process, the researcher explained the types of mould associated with mycotoxins to respondents using coloured pictures. This approach helped farmers better identify and confirm mycotoxin contamination in maize. All these strategies enrich the study’s insights for stakeholders in agri-food systems and contribute to a broader understanding of smallholder maize farmers in the Global South regarding their perceptions of mycotoxin contamination, the challenges they encounter in implementing control strategies, and the factors influencing their adoption of prevention and control measures. Based on the outcomes of this study, future research could empirically investigate the impact of climate change on mycotoxin contamination in maize and other crops. Additionally, similar studies could be conducted on crops such as cowpea, sorghum, and wheat, which are also susceptible to contamination and may pose health risks to humans and animals. Furthermore, research efforts could focus on the competencies and training needs of extension agents in disseminating mycotoxin-related information to farmers.

## 6. Materials and Methods

### 6.1. Study Area Description

**Figure 3:**
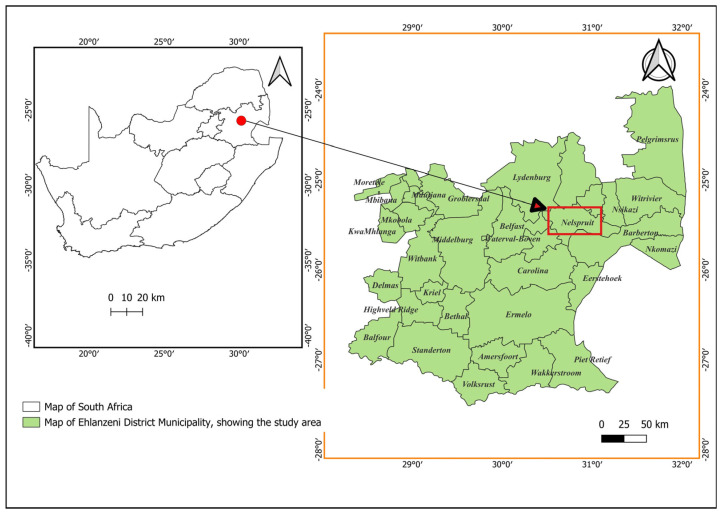
Map of South Africa showing Mpumalanga Province and Mbombela Local Municipality. Source: Sithole & Olorunfemi [49].

The study was conducted in the Mbombela Local Municipality of Mpumalanga province, situated in the north-eastern part of South Africa (Figure 3). The majority of smallholder farmers in the municipality grow maize alongside other grains, vegetables, and livestock, making the area well-suited for this study. Mbombela Local Municipality is located at the GPS coordinates −25.475, 30.969. The municipality’s maximum temperatures range from 25 °C in the western regions to 35 °C in the eastern parts [49]. This warm climatic condition favours the development of fungi responsible for mycotoxin production. According to [16], high levels of mycotoxin contamination have been identified in the Mpumalanga province. Furthermore, a recent study by Chukwudi et al. [6] reported high levels of maize mycotoxin contamination in the province. This made the province a suitable location for the study, thereby informing the selection of the study area.

### 6.2. Sampling

The study employed a quantitative research approach, using a cross-sectional survey design. A two-stage sampling procedure was adopted to select smallholder maize farmers in the study area. Firstly, a purposive selection of three (3) communities, namely Matsulu, Malekutu, and Salubinza, prominent for maize production in the municipality, was carried out. The second stage involved a random sample of 55, 61, and 36 smallholder maize farmers from Matsulu, Malekutu, and Salubinza, respectively. The exact number to select was computed using Slovin’s sample size formula at the 95% confidence level with a 5% margin of error. Thus, this gave a total of one hundred and fifty-two (152) maize farmers that were sampled for the study. Table 5 summarizes the sampling procedure for the study.

### 6.3. Data Collection and Analysis

Information was collected from respondents using structured questionnaires (see Supplementary Materials) administered and completed by trained enumerators during interviews with farmers. Informed consent was obtained from all participating farmers, and their involvement was entirely voluntary. Data collection was conducted in compliance with protocols and ethical principles outlined in the Declaration of Helsinki. Ethical clearance for this research was obtained from the University of Mpumalanga Ethics Committee under the reference number UMP/Shange/MAgric/2021. The survey instrument included sections that gathered information on the socio-demographic characteristics of smallholder maize farmers, their perceptions of the effects of mycotoxin contamination, the prevention and control practices they utilize, and the challenges they encounter in managing mycotoxin issues. Respondents were presented with a list of statements on the effect of mycotoxin contamination on income, health, and livelihoods. The responses were measured using a 5-point Likert scale of Strongly agree (5), Agree (4), Undecided (3), Disagree (2), and Strongly disagree (1). Also, respondents were asked to indicate mycotoxin prevention and control practices utilized from a list, which was measured as Utilized (1) and Not Utilized (0). The researcher further drew on Abegunde et al. [50] and Olorunfemi et al. [51] to compute a composite score for each respondent. The generated score was used as a proxy for the mycotoxin control utilization index for each farmer, which was then fitted as the dependent variable in a multiple linear regression to analyze the socio-demographic determinants of farmers’ utilization of mycotoxin prevention and control methods. Furthermore, a series of items was presented to the respondents, and they were asked to indicate the level of severity of these items as constraints on a 3-point severity scale: Very Severe (3), Moderately Severe (2), Not Severe (1). The data collected were analyzed using IBM SPSS version 28.0. Descriptive statistics (frequency counts, percentages, means, ranks, and charts) and inferential statistics (multiple linear regression) were used to analyze the collected data.

The explicit form of the model can thus be given as:

Y = *β*0 + *β*1X1 + *β*2X2 + _ _ _ _ _ _ _ _ _ + *β*nXn + e (1)

where Y is the maize farmer’s mycotoxin prevention and control utilization score; X is a vector of independent/explanatory variables; *β* is a vector of unknown parameters to be estimated; and e is an independent and normally distributed random error term. Table 6 shows the socio-economic variables fitted into the regression model.

## Figures and Tables

**Table 1:** Smallholder Maize Farmers’ Socio-economic Characteristics.

Variables	Frequency	Percentage (%)	Mean (SD)
Age			
≤30	33	21.7	45.61 (17.02)
31 to 60	83	54.6	
61 and above	36	23.7	
Gender			
Male	66	43.4	
Female	86	56.6	
Marital Status			
Not married	80	52.6	
Married	72	47.4	
Household Size			
1–5	75	49.3	5.8 (2.55)
6–10	69	45.4	
11 and above	8	5.3	
Formal Educational Level (Years of Schooling)			
No formal education	4	2.6	10.25 (4.07)
1–6	28	18.4	
7–12	83	54.6	
13 and above	37	24.3	
Maize Farming Experience (Years)			
1–5	48	31.6	11.41 (10.22)
6–10	45	29.6	
11–15	26	17.1	
16 and above	33	21.7	
Extension Visit			
Yes	118	77.6	
No	34	22.4	
Mycotoxin-related Training			
Yes	31	20.4	
No	121	79.6	
Farm Size			
Less than 1 ha	73	48.0	1.31 (1.14)
1 ha–5 ha	79	52.0	
Membership of Farmer Group			
Yes	71	46.7	
No	81	53.3	
Media Exposure			
1–2	31	20.4	
3 and above	121	79.6	

N = 152.

**Table 2:** Smallholder maize farmers’ perceived effects of mycotoxin contamination.

Perception Statements	Mean Score	Rank
Mycotoxin (moulds/fungi) contamination in maize is an important health hazard	4.25	5
Mycotoxin contamination often affects the change in taste, smell, and colour of maize grains	4.28	4
Consumption of mycotoxin-contaminated maize can result in a delay in child growth	3.35	11
The resistance of animals to diseases can be reduced by the consumption of mycotoxin-contaminated maize	3.34	12
The use of spoiled maize in making traditional beer should be avoided	3.37	10
Spoilt maize should not be sold or sent to the markets	4.36	3
Contaminated maize should not be used as animal feed, as this may lead to animal and human health problems	3.22	14
The use of mycotoxin-contaminated maize by humans can result in mild to severe disease complications, such as cancer	3.57	9
Mycotoxin-contaminated maize will be difficult to sell	4.41	2
Mycotoxin contamination will lead to a reduced market price of maize	4.43	1
The adverse health effects of mycotoxins range from acute poisoning to long-term effects such as immune deficiency and cancer	3.58	8
Mycotoxin contamination has negative impacts on livestock consuming contaminated feed	3.34	12
The poultry, fish, and dairy industries suffer income loss from mycotoxin contamination	3.19	15
Mycotoxin maize contamination may lead to household income loss	4.25	5
Mycotoxin may reduce farmers’ livelihood strategies	4.09	7

Mean scores were derived from strongly agree (5), agree (4), neutral (3), disagree (2) and strongly disagree (1).

**Table 3:** Constraints faced by farmers in preventing and controlling mycotoxin contamination in maize.

Challenges	Mean Score	Rank
	MS	
Inadequate awareness and training on mycotoxin contamination and control	2.50	1st
Inadequate access to adequate information on prevention and control	2.50	1st
Inadequate access to resistant seed variety	2.43	5th
Inadequate access to chemicals for seed treatment	2.39	7th
Climate change effects increase the risks of mycotoxin contamination	2.26	10th
Limited farm inputs for good agricultural practices	2.24	12th
Limited market access (grains stay longer in storage)	2.05	13th
Inadequate weed management in the fields	1.84	14th
Inadequacy of extension support services for the transfer of mycotoxin messages and control strategies	2.36	8th
Inadequate pest and disease management knowledge and measures	2.44	4th
High cost of improved seeds and mycotoxin-resistant maize cultivars	2.50	1st
Poor soil fertility leads to poor development of crops that are easily contaminated by mycotoxin fungal species	2.26	10th
Inadequate access to credit to improve farm production, technology, and improved prevention strategies	2.40	6th
Inadequate irrigation equipment for health and quality crop growth	2.29	9th

Note: Mean Score derived from not severe = 3, moderately severe = 2, and very severe = 1.

**Table 4:** Factors influencing maize farmers’ utilization of mycotoxin prevention and control methods.

Variables	Coeff.	Std Err.	T	*p* > t	VIF	Tolerance (1/VIF)
Gender	0.3102153	0.9264058	0.33	0.738	1.21	0.832685
Marital Status	0.1988488	0.9265849	0.21	0.830	1.27	0.811359
Household Size	0.1990081	0.1730405	1.15	0.252	1.12	0.896597
Farming Experience	−0.0919496	0.0498279	−1.85	0.067 *	1.48	0.673902
Education Level	−0.0077667	0.1294862	−0.06	0.952	1.59	0.630644
Extension Visits	1.970348	1.063584	1.85	0.066 *	1.13	0.884101
Mycotoxin Training	2.396084	1.163588	2.06	0.041 **	1.27	0.790061
Farm Size	0.6136892	0.3897622	1.57	0.118	1.13	0.888697
Membership of a Farm Group	0.6383667	0.896666	0.71	0.477	1.15	0.867763
Media Exposure	−0.9578758	0.3731593	−2.57	0.011 ***	1.14	0.877888
Awareness Index	0.3399412	1.1689752	2.01	0.046 **	1.14	0.879261
Perception Index	0.1590107	0.0524336	3.03	0.003 ***	1.19	0.842166
Constraints Index	−0.14488	0.0957417	−1.51	0.133	1.08	0.923078
Constant	30.18824	5.105546	5.91	0.000		
Mean VIF					1.22	
F	4.29					
Prob > F	0.0000					
R-Squared	0.2876					
Adj R-squared	0.2205					

Note: *, **, and *** mean 10%, 5% and 1% levels of significance, respectively.

**Table 5:** Summary of Sampling Procedure for the Study.

1st Stage: Purposive Sampling of Communities	Population of Registered Smallholder Maize Farmers in the Selected Communities According to the 2022 Database	2nd Stage: Proportionate Random Sampling of Study Participants Using the Slovin Formula
Matsulu	64	55
Malekutu	72	61
Salubinza	40	36
		Total Sample Size = 152

**Table 6:** Description of the socio-economic variables fitted in the regression model.

Variables	Description/Measurement	Variable Type	Hypothesized Sign
Gender	1 = Male, and 2 = Female	Categorical	Positive/negative
Marital status	1 = Married, 2 = Unmarried	Categorical	Positive/negative
Household size	Number of individuals in the household.	Continuous	Positive/negative
Farming Experience	Years spent cultivating maize	Continuous	Positive
Educational Level	Years spent in school	Continuous	Positive
Extension Visits	1 = Yes, 0 = Otherwise	Categorical	Positive
Participation in Mycotoxin-related Training	1 = If participated, 0 = Otherwise	Categorical	Positive
Farm Size	Total area of land used for maize cultivation in hectares	Continuous	Positive/negative
Membership of a Farmer Group	1 = If member of farmer group, 0 = Otherwise	Categorical	Positive
Media Exposure	The number of media outlets that farmers are exposed to in seeking and accessing information	Continuous	Positive
Awareness Index	PCA-generated index	Continuous	Positive
Perception Index	PCA-generated index	Continuous	Positive
Constraints index	PCA-generated index	Continuous	Negative

## Data Availability

The dataset generated and analyzed in this study is available from the corresponding author upon reasonable request from interested stakeholders due to participant confidentiality and because informed consent for unrestricted public data sharing was not obtained during data collection.

## Supplementary Materials

The following supporting information can be downloaded at: https://www.mdpi.com/article/10.3390/toxins18070289/s1, Data collection instrument: Questionnaire.
