# Supplementary material for: Mycotoxin Contamination in Smallholder Maize Production: Farmers’ Perceptions, Control Practices, and Influencing Factors in South Africa
Source: Toxins (Basel). 2026 Jun 30;18(7):289. doi: 10.3390/toxins18070289 (PMC13417403; doi:10.3390/toxins18070289)
Supplement: Supplementary file 1 [file toxins-18-00289-s001.zip › toxins-4351859-supplementary.pdf]

Questionnaire

Questionnaire number : ____________________

Instruction: Please Fill and Cross [X] In the appropriate place where necessary. Please complete all the items, unless advised differently.

Section A: Respondents' socio-economic characteristics

1. Age: Please indicate your age

	Years

2. Gender: Please indicate your gender

Female	
Male	

3. Marital Status: Please indicate your current marital status?

Married	
Unmarried	

4. Household size: Indicate the number of individuals you share basic utilities with within your house.

5. Farming Experience: Please indicate how long in years you have been in cultivating maize

	Years

6. Highest Education Attainment: Please indicate your years of schooling

7. Annual farming income: Indicate your average monthly income from your maize farming business

R

8. Secondary Occupation: Please indicate your secondary occupation apart from farming

9. Extension Visit: Do you receive farm visits and support from extension officers?

Yes	
No	

10. Agricultural Training: Have you participated in any mycotoxin related training?

Yes	
No	

11. Farm size: Indicate the total hectare of land you own which is used for maize cultivation

Acres

12. Membership of farmer group: Do you belong to any farmer group?

Yes	
No	

13. Media Exposure: Please indicate the number of media outlets you are exposed to.

Section B: Awareness of Mycotoxin (moulds/fungi) contamination in Maize

Kindly indicate your level of awareness of the following statement about mycotoxin maize contamination.

**Statements**	**Aware**	**Not Aware**
14. Mycotoxins(moulds/fungi) can be found in maize crops		

15. Maize crops can be contaminated by mycotoxins in the field and during storage		
16. High levels of rains during harvesting can increase the possibility of mycotoxin contamination in maize		
17. Maize should be harvested at the optimum stage of development		
18. Adequate drying of crops reduces mycotoxin contamination		
19. Insect infestation exposes maize crops to fungal infection		
20. Maize crops that are broken and bruised have an increased chances of being contaminated.		
21. Well dried grains can be less susceptible to contamination		
22. Mycotoxin can be easily spotted on maize		
23. Sorting of maize crops after harvesting can prevent mycotoxin contamination		
24. Untidy and improperly ventilated storehouse can predispose maize grains to mycotoxin contamination		
25. Fungal infections on maize grains may cause mycotoxins contamination		
26. Mycotoxin contamination is enhanced by poor storage conditions.		
27. The use of expired chemicals can predispose maize to mycotoxin contamination		
28. Mycotoxins are naturally occurring toxins found in maize		
29. Moulds grow on a variety of different crops and foodstuffs including maize		
30. Mycotoxin contamination is the food security and safety concern		

Section C: Perceived Effects of Mycotoxin Contamination

Kindly indicate your agreement or disagreement with the following statements on the effect of mycotoxin contamination in maize.

**Statements**	**Strongly Agree**	**Agree**	**Undecided**	**Disagree**	**Strongly Disagree**
31. Mycotoxin (moulds/fungi) contamination in maize is an important health hazard					

32. Mycotoxin contamination often affect the change in taste, smell and colour of maize grains					
33. Consumption of mycotoxin contaminated maize can result in a delay in child growth					
34. Resistance of animals to diseases can be reduced by the consumption of mycotoxin contaminated maize					
35. The use of spoilt maize in making traditional beer should be avoided					
36. Spoilt maize should not be sold or sent to the markets					
37. Contaminated maize should not be used as animal feed, as this may lead to animal and healthy problems					
38. The use of mycotoxin contaminated maize by humans can result in mild to severe disease complication such as cancer					
39. Mycotoxin contaminated maize will be difficult to sell					
40. Mycotoxin contamination will lead to the reduced market price of maize					
41. The adverse health effects of mycotoxins range from acute poisoning to long-term effects such as immune deficiency and cancer					
42. Mycotoxin contamination has negative impacts on livestock consuming contaminated feed					
43. The poultry, fish and dairy industries suffer income loss from mycotoxin contamination					
44. Mycotoxins can enter the food chain in the field, at storage, or at postharvest					
45. Crops with large amounts of mycotoxins often must be destroyed					
46. Mycotoxin maize contamination may lead to household income loss					
47. Mycotoxin may reduce farmers' livelihoods strategies					

Section D: Utilization of Preventive and Control Practices

Indicate whether the following practices are utilised on your farm or not.

**Practices**	**Utilized**	**Not Utilized**
48. Planting of healthy and quality seeds		
49. Use of good field management practices		
50. Adequate drying of maize before storage		
51. Proper sorting of harvested grains to prevent fungal development		
52. Storage of maize in clean and well-ventilated stores		
53. Proper handling of maize when transporting for market purposes		
54. Use of crop rotation		
55. Timely and adequate control of pest and diseases in maize farm		
56. Seed treatment with chemical fungicide before planting		
57. Removal of stubble from previous crops		
58. Treatment of seeds with biological control		
59. Prevention of damage to grains during harvesting and transportation		
60. Fumigation of storage rooms before maize storage		
61. Cleaning harvested grains before storage		
62. Use of antimicrobial agents to prevent fungal activity on maize		
63. Harvesting maize at the optimum stage of development		
64. Plant resistance cultivars of maize		
65. The use of detoxifying agents (enzymes and binders) on maize		
66. The use of genetic modification cultivars		
67. Others:		

Section E: Challenges faced in the prevention and Control of Mycotoxins Contamination

Indicate the level of severity of challenge you face as it relates to preventing and controlling mycotoxin contamination on your maize farm

**Challenges**	**Very Severe**	**Moderately Severe**	**Not Severe**
68. Inadequate awareness and training on mycotoxin contamination and control			
69. Lack of access to adequate information on prevention and control			
70. Inadequate access to resistant seed variety			
71. Inadequate access to chemicals for seed treatment			
72. Climate change effects increase risks of mycotoxin contamination			
73. Limited farm inputs for good agricultural practices			
74. Limited market access (grains stay longer in storage)			
75. Lack of weed management in the fields			
76. Inadequacy of extension support services for transfer of mycotoxin messages and control strategies			
78. Lack of pest and disease management knowledge and measures			
79. High cost of improved seeds and mycotoxin resistant maize cultivars			
80. Poor soil fertility leads to poor development of crops that are easily contaminated by mycotoxin fungal species			
81. Lack of access credit to improve farm production, technology, and improved prevention strategies			
82. Lack of irrigation equipment for health and quality crop growth			
83. Others:			
